# Learning curve of transanal minimally invasive surgery for rectal neoplasm

**DOI:** 10.3389/fonc.2025.1545589

**Published:** 2025-03-25

**Authors:** Xingwang Li, Shaoqing Guo, Kunhou Yao, Zheng Ge, Yuewei Li, Junhong Hu, Hongping Xia

**Affiliations:** ^1^ Department of General Surgery, The First Affiliated Hospital of Xi’an Jiaotong University, Shaanxi, China; ^2^ Department of General Surgery, Huaihe Hospital Affiliated to Henan University, Henan, China; ^3^ Department of Colorectal and Anal Surgery, First Affiliated Hospital of Zhengzhou University, Henan, China; ^4^ Zhongda Hospital, School of Medicine, Advanced Institute for Life and Health, Southeast University, Jiangsu, China

**Keywords:** transanal minimally invasive surgery, learning curve, rectal neoplasm, safety, transanal endoscopy

## Abstract

**Objectives:**

The field of view through transanal endoscopic provides new treatment approaches for solving complex clinical problems. TAMIS belongs to single-port endoscopic surgery, and the operation is complex. Analyzing the learning curve of TAMIS aims to facilitate its better clinical promotion.

**Methods:**

A retrospective cohort study analyzed the clinical data of 58 patients who underwent TAMIS by the same surgeon from January 2018 to October 2024. The learning curve of TAMIS was obtained using the cumulative sum (CUSUM) analysis, and the optimal number of surgeries was determined based on the peak value of the curve, Clinical indicators such as operative time, intraoperative blood loss, positive rate of circumferential margin, length of postoperative hospital stay, and incidence of postoperative complications were compared at different stages.

**Results:**

All 58 patients successfully underwent TAMIS. The optimum curve equation was y=0.016*x* ^3^-2.0556*x* ^2^+67.240*x*-150.103, *R* ^2^ = 0.950, *P*<0.05. According to the peak value of the curve, 22 cases were determined as the minimum cumulative required cases for surgeons to cross the TAMIS learning curve. 58 cases were divided into two groups: the learning improvement group (Pre-proficiency) of the first 22 cases, and the proficiency group (Post-proficiency) of the latter 36 cases. Compared with Pre-proficiency stage, the Post-proficiency stage had shorter surgery duration, less intraoperative blood loss, and shorter length of postoperative hospital stay (*P*<0.05). There was no statistically significant difference in the observation indicators including positive rate of circumferential margin and incidence of postoperative complications between the two groups (*P*>0.05).

**Conclusions:**

The learning curve of TAMIS can be divided into Pre-proficiency stage and Post-proficiency stage. 22 surgeries may be the number of surgeries required to cross the TAMIS learning curve.

## 1 Introduction

With the development of surgical techniques, new endoscopic techniques and surgical approaches continuously emerge. Transanal endoscopic surgery (TAES) refers to surgical procedures using endoscopic instruments for transanal access. including Transanal endoscopic microsurgery (TEM), Transanal total mesorectal excision (taTME), and Transanal minimally invasive surgery (TAMIS) (1). TEM was first invented by German scholar Gerhard Buess in 1983 as an operating platform, which was primarily used for treating of rectal polyps and early rectal cancer (2). Sylla reported the use of TEM platform in laparoscopic assisted taTME surgery in 2009 (3). Subsequently, Albert invented a soft single-port endoscopic platform for rectal polypectomy surgery in 2010, named TAMIS (4). Due to its minimal trauma and fast postoperative recovery, TAMIS is widely applied in treating rectal neoplasm and early rectal cancer, and has achieved good clinical application results (5, 6). The learning curve (LC), also known as the experiential curve, LC represents the process in which producers continuously improve their work efficiency through the accumulation of learning and experience. In 1936, Wright described the learning curve in the manufacturing industry (7). Afterwards, scholars conducted extended research on the model, Mainly by modifying the parameters of the model to adapt to different situations, it reflects the process of people learning a new thing. In the field of clinical medicine, learning curve can help clinical doctors master the technology faster and better (8, 9). This study retrospectively analyzed the clinical data of 58 TAMIS patients treated by the same surgical team in our center from January 2018 to October 2024. The CUSUM analysis method was used to explore the learning curve, aiming to promote TAMIS specialist training and provide clinical promotion reference.

## 2 Materials and methods

### 2.1 Patient characteristics

A retrospective cohort study was performed to analyze the clinical data of patients who underwent TAMIS in the Department of General Surgery at the First Affiliated Hospital of Xi’an Jiaotong University from January 2018 to October 2024. Inclusion criteria were as follows: ① Colonoscopy clearly shows rectal neoplasm, with a diameter ≤ 5cm and located 2-10 cm for the anal verge; ② No history of rectal surgery, and no radiation therapy or chemotherapy; ③ Preoperative enhanced computed tomography (CT) or magnetic resonance imaging (MRI) showed no distant organ metastasis, surrounding tissue invasion, metastatic lymph node lesions, and the neoplasm did not invade the rectal intrinsic muscle layer; ④ The clinical pathological data is complete. Exclusion criteria included: ①-Contraindications for laparoscopic surgery; ② Multiple primary tumor lesions; ③ Inability to tolerate surgery or anesthesia. After screening according to the inclusion and exclusion criteria, a total of 58 patients were included in the study.

This study adhered to the Consolidated Standards of Reporting Trials Statement (CONSORT), Patient enrolment was initiated after obtaining written informed consent. All enrolled patients were operated on by the same surgeon, who had received systematic training in laparoscopic surgery for colorectal cancer, possessing extensive experience in laparoscopic colorectal cancer surgery.

### 2.2 Data collection

The main observation indicators are operative time and intraoperative blood loss. The secondary observation indicators include the patient’s age, gender, body mass index (BMI), American Society of Anesthesiologists (ASA) grade, tumour location, tumour distance from the anal verge, tumour size, pathological type, positive rate of circumferential margin, incidence of postoperative complications, and length of postoperative hospital stay.

### 2.3 Surgical technique

**Figure 1:**
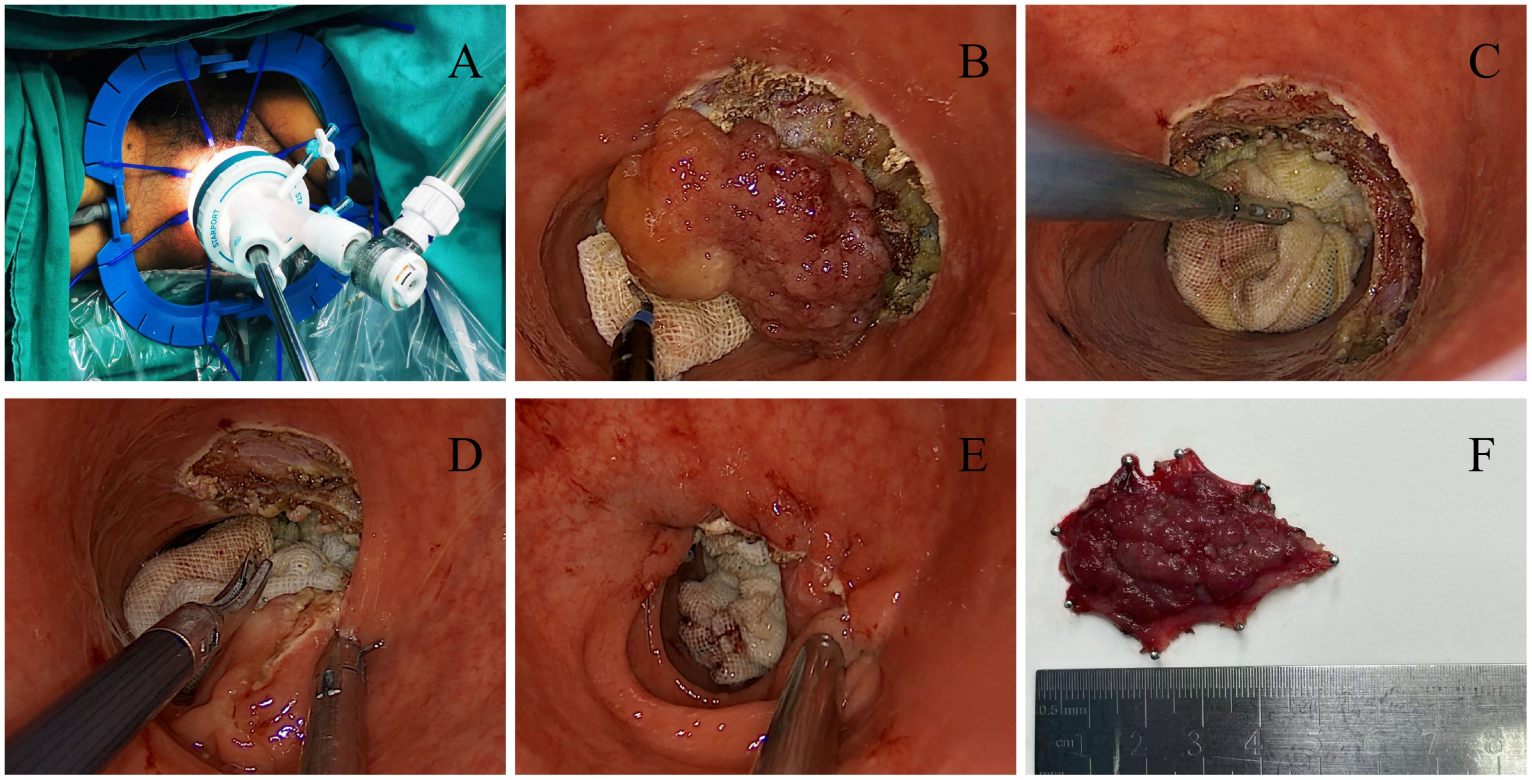
**(A)** Insert a soft single port; **(B)** Resection along the edge of the tumor; **(C)** Surgical wound after complete tumor resection; **(D)** Suture and close the surgical wound; **(E)** Surgical wound after surgery; **(F)** Postoperative specimen.

TAMIS is a completely transanal single-port endoscopic surgery. The steps are as follows: fully expand the anus and install the anal Lone-star; rinse the rectal cavity with iodine solution; explore the tumor’s location and size; Insert a soft transanal single-port operating platform; place gauze strips into the tumor’s proximal segment to block the upper intestinal tract and prevent intestinal content overflow. Next, create a space for endoscopic manipulation, insert endoscopic instruments, and resect along the tumor edge until complete resection. Use 2-0 absorbable suture to close surgical wound, carefully achieve hemostasis, place the anal canal, and end the surgery, as shown in **Figure 1**.

Postoperatively, the rectal tube is typically removed when patients begin passing gas, usually 2-3 days after surgery. Patients are then advised to start a liquid diet. When normal bowel movements resume and fever/pain symptoms disappear, the patient meets discharge criteria.

### 2.4 CUNSUM analysis

All surgical cases were sorted by surgical dates. Operative time referred to the duration from establishing the anal single-port operation platform to completely closing the surgical wound. The CUSUM analysis method was used to plot the learning curve, The formula is: Here, X*i* represents the operative time (or intraoperative blood loss), U represents the average operative time (or average intraoperative blood loss), and represents the number of surgeries, The CUSUM value reflects the cumulative sum of the difference between each patient’s operative time (or intraoperative blood loss) and the average value, as well as difference across all previous cases. The initial CUSUM value is 0.

The y-axis represents the CUSUM value of operative time (or intraoperative bleeding), and the x-axis represents the number of patient cases.GraphPad Prism 8 software is utilized to draw and fit the learning curve scatter plot. The equation with a fitting coefficient R^2^ value closer to 1 and *P* < 0.05 is selected as the best fitting curve equation to plot the fitting curve, where the vertex decline point indicates the number of surgical cases completing the learning period. According to the peak value, the learning curve is divided into two stages: the Pre-proficiency stage and the Post-proficiency stage.

### 2.5 Statistical analysis

Statistical analysis is performed using SPSS 24.0. For metric data following a normal distribution, the t-test is used for inter-group comparison. Non-normally distributed metric data are represented as M(Q1, Q3). Mann Whitney U test is used for inter group comparison. Count data are expressed as a percentage(%), and group comparison are performed using the χ2 test or Fisher’s exact probability test. *P* < 0.05 is defined as a statistically significant difference.

## 3 Results

### 3.1 Patient characteristics

58 patients with rectal neoplasm who underwent TAMIS at the First Affiliated Hospital of Xi’an Jiaotong University from January 2018 to October 2024 were included. There were 30 males (51.72%) and 28 females (48.28%). Including 9 cases of tubular adenoma (15.52%), 10 cases of Tis (17.24%), 7 cases of moderately differentiated adenocarcinoma (12.07%), 11 cases of well differentiated adenocarcinoma (18.97%), 6 cases of gastrointestinal stromal tumor (GIST) (10.34%), 3 cases of neuroendocrine tumors (5.17%), 3 cases of granulomas (5.17%), 2 cases of inflammatory hyperplasia (3.45%), 2 cases of cap polyps (3.45%), 2 cases of cystic enteritis (3.45%), and 1 case of melanoma (1.72%). All surgeries were successfully completed, with no conversion to open surgery, no perioperative deaths, and no serious complications such as anastomotic fistula after surgery. All patients’ postoperative pathological circumferential margins were negative.

### 3.2 Learning curve analysis

**Figure 2:**
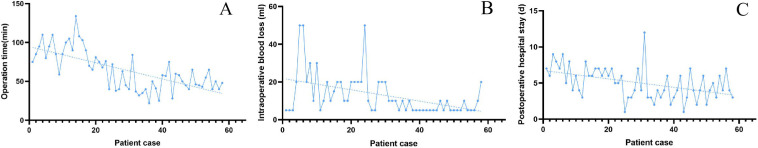
**(A)** Scatter plot and trend plot of operative time; **(B)** Scatter plot and trend plot of intraoperative blood loss; **(C)** Scatter plot and trend plot of postoperative hospital stay.

The scatter plot trend indicated that both operative time and intraoperative blood loss decrease as the number of cases increased. The average operative time was 64.07 minutes, the average blood loss was 13.02 mL, and the average postoperative hospital stay was 5.02 days, as shown in **Figure 2**.

**Figure 3:**
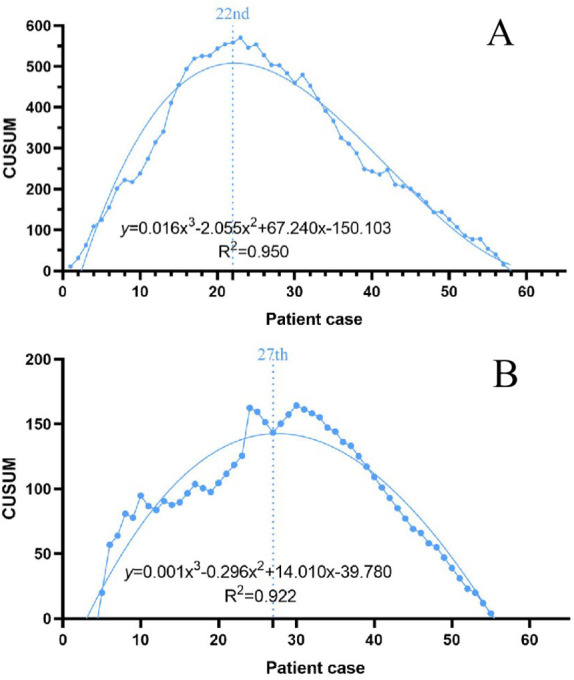
**(A)** Learning curve of operative time; **(B)** Learning curve of intraoperative blood loss.

Using GraphPad Prism 8, scatter plots of CUSUM values for operative time and intraoperative blood loss were drawn and fitted. The fitting curve equation for operative time was y=0.016x^3^-2.0556x^2^+67.240x-150.103, R^2^ = 0.950(*P*<0.05). The fitting curve equation for Intraoperative blood loss was y=0.001x^3^-0.296x^2^+14.010x-39.780, R^2^ = 0.922(*P*<0.05). The R^2^ of the fitting equation for operative time was closer to 1. y=0.016x3-2.0556x2 + 67.240x-150.103 was selected as the optimal learning curve equation. The curve peaked at the 22nd cumulative case, Based on the trend, the learning curve is divided into two stages, Pre-proficiency stage and Post-proficiency stage. The 22nd case is the minimum cumulative number of operative cases required to cross the learning curve, as shown in **Figure 3**.

### 3.3 Comparison of Before and After the Completion of LC

All 58 surgeries were successfully completed, The postoperative pathology of 58 patients included 28 adenocarcinoma cases, and all circumferential margins were negative. To verify the results, general patient information between the Pre-proficiency stage group and the Post-proficiency stage group was compared. There were no statistically significant differences (*P* > 0.05) in age, gender, BMI, ASA grade, tumour size, tumour distance from the anal verge, or tumour location between the two groups. However, significant statistical differences (*P* < 0.05) existed in operative time, intraoperative blood loss, and postoperative hospital stay. The operative time and postoperative hospitalization time in the Pre-proficiency stage group were significantly longer than those in the Post-proficiency stage group; The intraoperative blood loss in the Pre-proficiency stage group was significantly higher than that in the Post-proficiency stage group, as shown in **Table 1**.

**Table 1:** Comparison of materials between the Pre-proficiency group and the Post-proficiency group.

	Pre-proficiency (*n*=22)	Post-proficiency (*n*=36)	*t/χ^2^*	*p*
Age (yr)	64.18 ± 12.32	57.53 ± 13.56	1.876	0.066
Gender			0.770	0.380
Male	13 (59.1%)	17 (47.2%)		
Female	9 (40.9%)	19 (52.8%)		
BMI (kg/m^2^)	25.68 ± 3.29	26.35 ± 2.90	-1.507	0.141
ASA grade			0.003	0.955
I	9 (40.9%)	15 (41.7%)		
II	13 (59.1%)	21 (58.3%)		
Tumour size (cm)	3.04 ± 0.99	2.25 ± 1.05	1.775	0.082
Distance of the tumours from the anal verge (cm)	5.00 ± 2.31	4.67 ± 1.96	0.564	0.576
Location			2.678	0.261
Anterior	10 (45.5%)	9 (25.0%)		
Posterior	6 (27.3%)	12 (33.3%)		
Lateral	6 (27.3%)	15 (41.7%)		
Pathological type			0.293	0.588
Adenocarcinoma	10 (45.5%)	19 (52.8%)		
Non adenocarcinoma	12 (54.5%)	17 (47.2%)		
Operative time (min)	89.45 ± 17.96	48.56 ± 14.61	9.014	<0.001
Intraoperative blood loss (ml)	18.41 ± 12.67	9.72 ± 8.61	2.480	0.008
Postoperative hospital stay (d)	6.41 ± 1.56	4.17 ± 2.10	4.635	<0.001

## 4 Discussion

Currently, the development of colorectal surgery is trending toward greater standardization and minimal invasive. Laparoscopic technology has been extensively developed and applied in gastrointestinal surgery, extending into multiple domains like robotic endoscopy, single-port endoscopy, and transanal endoscopy (10). Transanal endoscopic techniques, mainly represented by TaTME, has become a hot topic in recent colorectal surgery discussions, The technique has achieved significant progress in both technical innovation and clinical research (11). TAMIS as a transanal endoscopic surgical method, is particularly suitable for rectal adenomas and T1-stage rectal cancer with favorable pathological features (12). TAMIS is widely implemented in large medical centers, and there are very few reports on its learning curve.

A total of 58 patients were included in this study, The results showed that the operative time, intraoperative blood loss, and postoperative hospitalization time exhibited a decreasing trend as the number cases increased, The CUSUM fitting curve peaked at the 22nd accumulated surgical case, indicating this as the minimum number of surgeries required to cross the learning curve. This result is consistent with a retrospective study by Lee L (13). Lee L conducted a study with R1 resection as the main observation indicator, revealing that TAMIS achieved acceptable R1 resection in 14-24 cases, while achieving shorter operation time. Park SS from South Korea used simulator training to study the learning curve of TAMIS, and the results showed that the ideal surgical effect could be achieved when the training frequency reached 15-20 times (14). There is also a study from the Clermonts SHEM team in the Netherlands (15), which conducted a prospective study on TAMIS performed by two surgeons. The observation indicators were whether the margin was positive, intraoperative blood loss, and operation time. The results showed that at least 18-31 surgical cases were needed to achieve satisfactory TAMIS outcomes, which is also consistent with our results.

At present, the most questioned and controversial aspect of TAMIS is its safety and oncological efficacy. Among them, safety is mainly manifested in intraoperative and postoperative bleeding and Carbon Dioxide Metabolism. Our research results showed that none of the 58 patients had positive surgical margins or serious complications like bleeding, perforation, or anastomotic leakage, which may be related to the small sample size in our study. As for Carbon Dioxide Metabolism, Edward have provided a detailed introduction, key contributing factors include high transanal pneumoperitoneum pressure and venous vessel rupture/bleeding, which allow CO2 to enter venous vessels. The sudden drop in end-to-end carbon dioxide partial pressure serves as critical early detection signal for carbon dioxide metabolism issues. Early detection and treatment are very important to avoid serious adverse events as much as possible (16). Concerning oncological outcomes, Lee L described the oncological follow-up of 200 TAMIS surgeries, with inclusion criteria for benign tumors that cannot be removed under endoscopy or early-stage T1 rectal cancer, including 90 benign tumors and 110 malignant tumors. The results showed that the overall positive rate of surgical margin was 7%, and the incidence of postoperative complications was 11%, including bleeding (9%) and urinary retention (4%). During an average follow-up of 14.4 months, 6% experienced local recurrence and 2% experienced distant organ metastasis. The disease-free survival (DFS) for patients with rectal adenocarcinoma at 1, 2, and 3 years were 96%, 93%, and 84%, respectively (17). Garoufalia Z included 7 observational studies on the surgical outcomes of TAMIS and TEM in a meta-analysis. The results showed that TAMIS and TEM had similar surgical outcomes and specimen quality, but TAMIS had lower readmission rates and overall complication rates, highlighting the advantages of TAMIS on soft platforms over TEM on hard platforms (11). The results were consistent with Lee L (18). For the treatment of other rectal tumors, such as neuroendocrine tumors, GIST, etc., TAMIS also has good effects (19, 20).

This study characterizes the learning curve of TAMIS, aiming to provide reference for colorectal surgeons and theoretical basis for its broader clinical application and promotion. However, our study is only based on the experience of one surgeon regarding the LC of TAMIS, LC of 22 cases should be confirmed by other studies investigating the LC of other surgeons. We believe that with the improvement of endoscopic technology and the advancement of medical equipment, transanal endoscopic surgery will demonstrate advantages in managing a broader spectrum of colorectal diseases. However, our single-center research also has limitations such as a small sample size, diverse pathological types, and lack of long-term oncological follow-up data. Therefore long-term oncological effects still require further validation through high-quality clinical studies with large sample sizes.

## Data Availability

The original contributions presented in the study are included in the article/supplementary material. Further inquiries can be directed to the corresponding authors.
